# Sagittal femoral bowing contributes to distal femoral valgus angle deviation in malrotated preoperative radiographs

**DOI:** 10.1186/s12891-022-05542-z

**Published:** 2022-06-15

**Authors:** Yasuhiko Kokubu, Shinya Kawahara, Satoshi Hamai, Yukio Akasaki, Hidetoshi Tsushima, Kenta Momii, Yasuharu Nakashima

**Affiliations:** 1 Department of Orthopaedic Surgery, Graduate School of Medical Sciences, Kyushu University, 3-1-1 Maidashi, Higashi-ku, Fukuoka, 812-8582 Japan; 2 Department of Medical-Engineering Collaboration for Healthy Longevity, Kyushu University, 3-1-1 Maidashi, Higashi-ku, Fukuoka, 812-8582 Japan; 3 Emergency and Critical Care Center, Kyushu University, 3-1-1 Maidashi, Higashi-ku, Fukuoka, 812-8582 Japan

**Keywords:** Distal femoral valgus angle, Femoral shaft bowing, Malrotation, Total knee arthroplasty, Whole-leg radiography

## Abstract

**Background:**

The coronal whole-leg radiograph is generally used for preoperative planning in total knee arthroplasty. The distal femoral valgus angle (DFVA) is measured for distal femoral bone resection using an intramedullary guide rod. The effect of coronal and sagittal femoral shaft bowing on DFVA measurement in the presence of malrotation or knee flexion contracture has not been well reported. The objectives of this study were: (1) to investigate the effects of whole-leg malrotation and knee flexion contracture on the DFVA in detail, (2) to determine the additional effect of coronal or sagittal femoral shaft bowing.

**Methods:**

We studied 100 consecutive varus and 100 valgus knees that underwent total or unicompartmental knee arthroplasty. Preoperative CT scans were used to create digitally reconstructed radiography (DRR) images in neutral rotation (NR, parallel to the surgical epicondylar axis), and at 5° and 10° external rotation (ER) and internal rotation (IR). The images were also reconstructed at 10° femoral flexion. The DFVA was evaluated in each DRR image, and the angular variation due to lower limb malposition was investigated.

**Results:**

The DFVA increased as the DRR image shifted from IR to ER, and all angles increased further from extension to 10° flexion. The DFVA variation in each position was 1.3° on average. A larger variation than 2° was seen in 12% of all. Multivariate regression analysis showed that sagittal femoral shaft bowing was independently associated with a large variation of DFVA. Receiver operating characteristic analysis showed that more than 12° of sagittal bowing caused the variation.

**Conclusion:**

If femoral sagittal bowing is more than 12°, close attention should be paid to the lower limb position when taking whole-leg radiographs. Preoperative planning with whole-leg CT data should be considered.

## Background

During total knee arthroplasty (TKA), surgeons have traditionally tried to place the femoral component perpendicular to its mechanical axis [1, 2]. In preoperative planning, the whole‐leg radiograph is used to measure the angle between the femoral mechanical axis and the anatomical axis of the distal femur in the coronal plane, to facilitate the use of the intramedullary guide rod. For accuracy, the coronal whole-leg radiograph must be evaluated in neutral rotation (NR). However, radiographs of varus knees are generally taken in a slightly externally rotated (ER) position, while valgus knees are usually examined in a slightly internally rotated (IR) position. Flexion contracture is also common in severely deformed knees. However, few studies have described in detail the effects of lower limb malrotation and knee flexion on the distal femoral valgus angle (DFVA) for TKA preoperative planning.

Morphological features of the femur, such as coronal and sagittal femoral shaft bowing, may increase the effect of whole-leg malrotation and knee flexion contracture on the measured DFVA. Femoral shaft bowing has been associated with Asian ethnicity, age, and the progression of knee osteoarthritis (OA) [3]. Coronal femoral shaft bowing greater than 5° has been described as a risk factor for postoperative malalignment [4]. Sagittal femoral shaft bowing has been shown to cause increased femoral component flexion in TKA [5]. However, it is not clear how coronal and sagittal femoral shaft bowing affects the measurement of the DFVA when there is malrotation.

The objectives of this study were to use three-dimensional (3D) computer simulations, first, to investigate the effects of whole-leg malrotation and knee flexion contracture on the DFVA and, second, to determine the additional effect of coronal or sagittal femoral shaft bowing.

## Materials and methods

### Data acquisition

Consecutive patients with varus or valgus deformity who underwent TKA or unicompartmental knee arthroplasties in our institution were included in the study. Patients with any history of osteotomy, fracture, or arthroplasty of the hip or knee joint were excluded. We recruited 100 varus and 100 valgus knees. The varus knees were recruited between April 2019 and June 2021, and the valgus knees were recruited between April 2012 and June 2021. All the patients were Japanese and provided informed consent before participation. The local Institutional Review Board approved the study (No.2020–204).

Varus or valgus alignment is based on the hip–knee-ankle (HKA) angle (the angle between the mechanical axes of the femur and tibia). The HKA angle was measured with anteroposterior whole-leg standing radiographs using Fuji-film OP-A software (Fujifilm, Co., Ltd, Tokyo, Japan).

Preoperative transverse CT scans (Aquilion ONE; Canon Medical Systems Corporation, Tochigi, Japan) of the lower extremity (including hip and ankle joints) were taken in all patients at 1.25 mm intervals and 1.25 mm thickness with a field of view of 400 and 1.375 pitch. The patients, supine on the scanning table, were instructed to naturally extend their affected knee without any feeling of internal or external rotation. The CT images were acquired as Digital Imaging and Communications in Medicine (DICOM) format data from the CT system server.

### 3D coordinate system definition and DRR image reconstruction of the femur

**Fig. 1:**
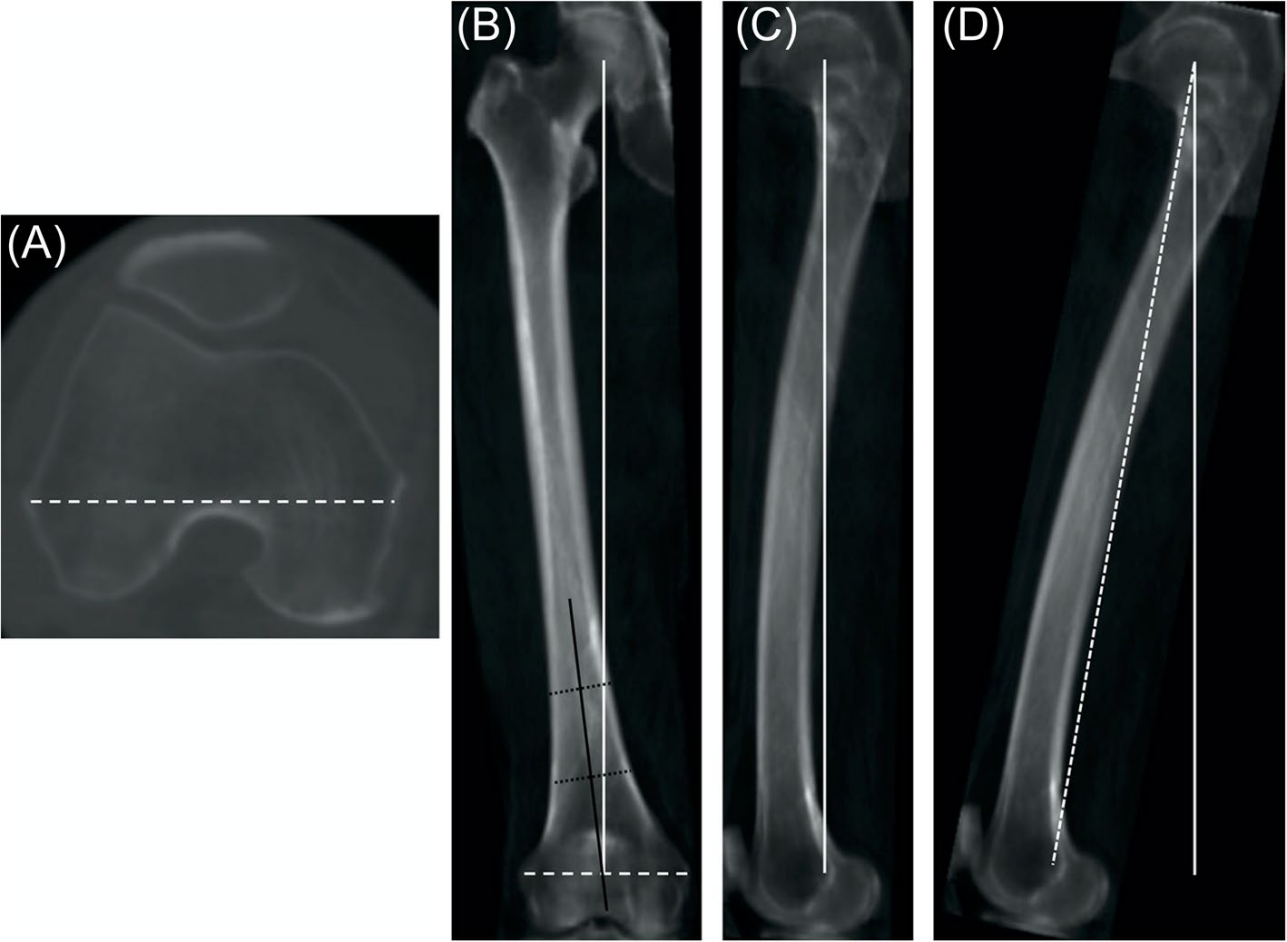
**A** The surgical epicondylar axis (SEA). **B**, **C** Coronal and sagittal DRR images. White solid line, white dashed line and black solid line indicate the mechanical axes of femur, the SEA and the distal femoral anatomical axis, respectively. **D** 10° flexed position in the sagittal plane

The DICOM data sets were imported into 3D planning software (3D template; Kyocera, Osaka, Japan). The femoral mechanical axis was defined as the line connecting the femoral head center and the midpoint of the surgical epicondylar axis (SEA; a line connecting the tip of the lateral epicondyle and the medial epicondylar sulcus, Fig. 1A) [6]. The 3D femoral coordinate system was defined as follows: the femoral head center and the femoral mechanical axis were defined as the origin and proximal–distal axis. The coronal plane was defined as the plane including the femoral mechanical axis and the SEA (Fig. 1B).

**Fig. 2:**
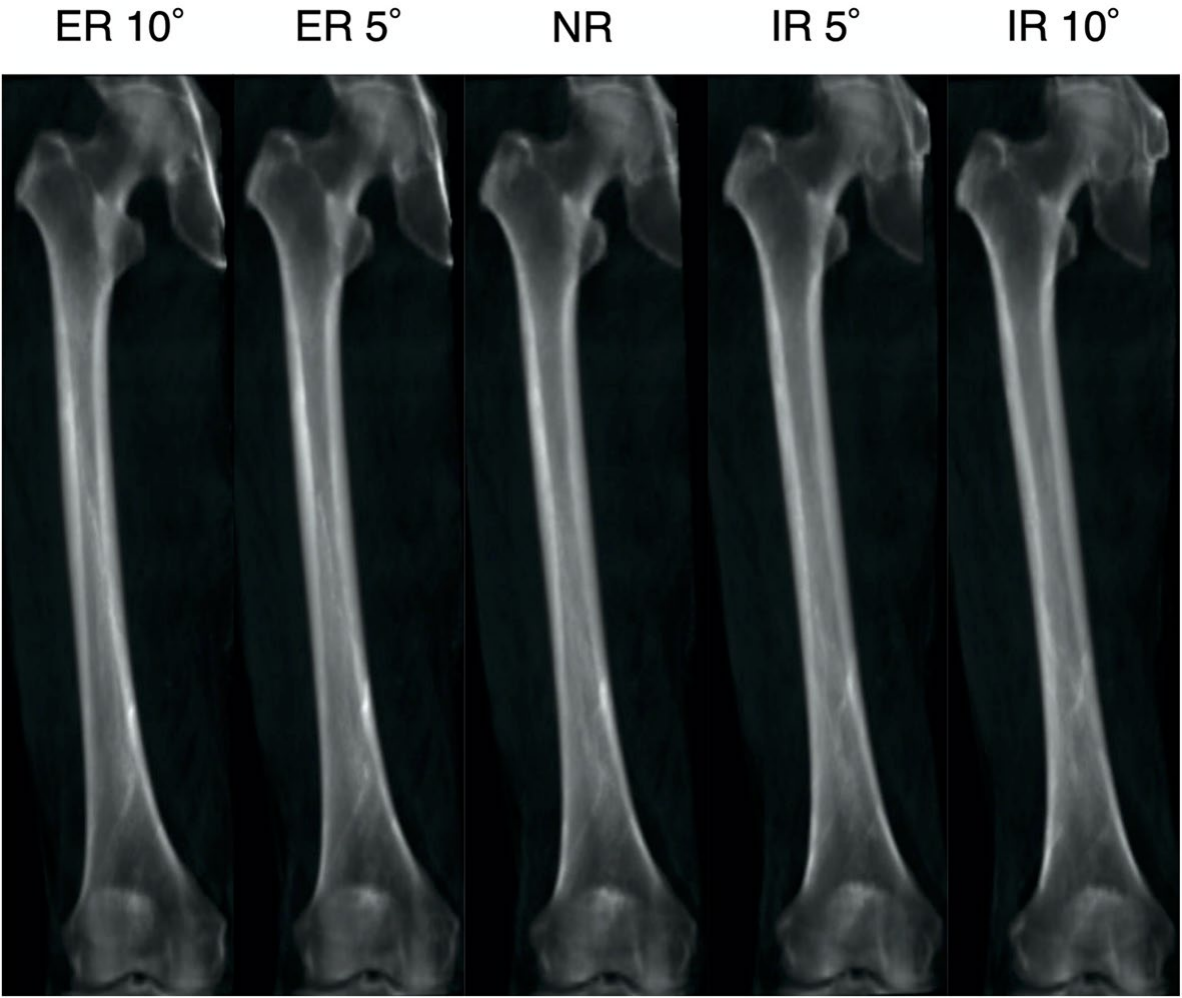
The digitally reconstructed radiography (DRR) images parallel to the SEA (NR), 5° and 10° relative to the SEA, respectively, were reconstructed. ER, external rotation; IR, internal rotation; NR, neutral rotation; SEA, surgical epicondylar axis

First, the digitally reconstructed radiography (DRR) coronal image based on the 3D femoral coordinate system described above was defined as the NR femoral radiograph. Then, DRR images at 5° and 10° ER and IR relative to the SEA were reconstructed (Fig. 2). Second, DRR images with NR and 5° and 10° of ER and IR were also reconstructed with 10° femoral flexion. At 10° femoral flexion, the tibia would also be almost 10° flexed. Therefore, this position is considered to replicate approximately 20° of knee flexion contracture. The DRR image with neutral flexion and rotation was reconstructed in the sagittal plane to measure the sagittal femoral shaft bowing angle (Fig. 1C).

### Measurement of the distal femoral valgus angle and the femoral shaft bowing angle

The DFVA was defined as the valgus angle between the femoral mechanical axis and the distal femoral anatomical axis (the axis connecting the femoral shaft centers at 5 cm and 10 cm proximal to the midpoint of the SEA) [7, 8]. It was measured in each DRR image (Fig. 1B), and the difference relative to the neutral flexion and rotation was calculated. The DFVA variation due to the lower limb position was also calculated as the difference between the maximum and minimum measured angles in each DRR image.

**Fig. 3:**
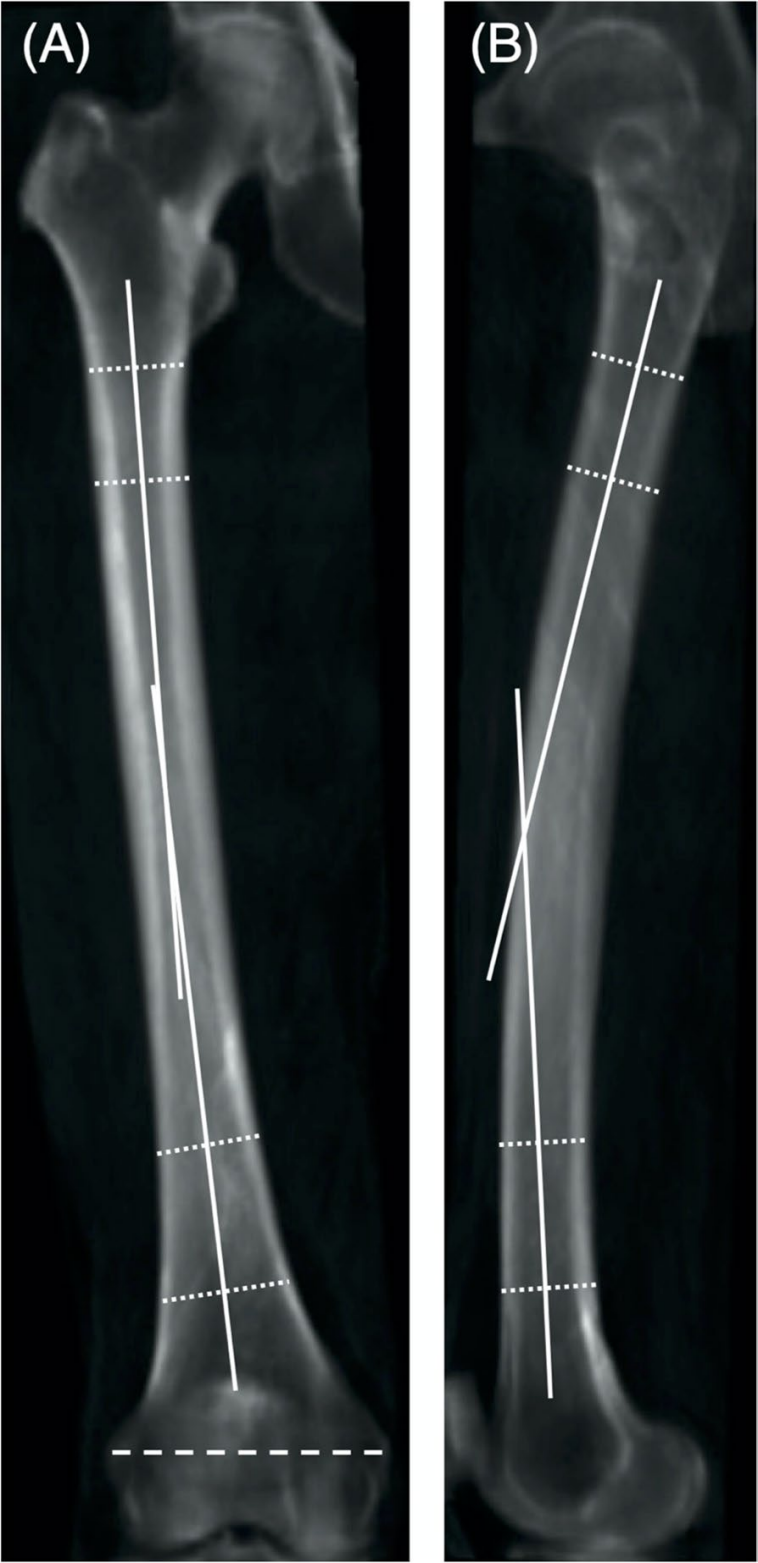
Coronal (**A**) and sagittal (**B**) femoral shaft bowing angles were defined as the acute angles between the proximal and distal femoral anatomical axes

The proximal femoral anatomical axis was defined as the axis connecting the femoral shaft centers at the lower end of the lesser trochanter and 5 cm distal to the lesser trochanter [7]. The femoral shaft bowing angle was measured on the NR, coronal and sagittal DRRs, as the angle between the proximal and femoral anatomical axes (Fig. 3) [8, 9].

### Statistical analysis

The Wilcoxon rank-sum test was used to compare the continuous parameters. The Chi-square test was used to compare categorical parameters between the varus and valgus HKA angle groups.

The DFVA at neutral and 10° of flexion in each rotational limb position were compared by paired t-test. Statistical significance was set at *p* < 0.05. A variation of 2° or more in DFVA was defined as a "large variation" based on the previous reports [10, 11]. A multivariate analysis was conducted to investigate the factors that cause a large variation in the DFVA between each limb position. Before the analysis, univariate analysis was performed for factors with large variation, and factors with *p* < 0.20 were selected. Multivariate analysis was conducted using those factors to identify the independent influence of each factor. These analyses were performed for varus and valgus knees, respectively.

According to Bao et al., there is no significant correlation between coronal and sagittal shaft bowing [12]. Therefore, the coronal and sagittal shaft bowing were analyzed as independent parameters. Statistical analyses were performed using JMP statistical analysis software (version 15.0; SAS Institute, Cary, NC, USA). To evaluate the intraobserver and interobserver reproducibility, measurements were performed twice by one examiner (YK) and once by another examiner (RK) on the study group. The intraclass correlation coefficient and the interclass correlation coefficient were good (0.80 and 0.75, respectively) for DFVA measurements, good (0.85 and 0.81, respectively) for coronal bowing measurements, and good (0.82 and 0.78, respectively) for sagittal bowing measurements.

## Results

Patient demographic and radiographic data are presented in Table 1. The BMI was higher in the valgus knee group significantly. Coronal and sagittal shaft bowing angles were significantly higher in the varus knees.

**Table 1:** Patient demographic and radiographic data

Parameters	Valgus (*n* = 100 knees)	Varus (*n* = 100 knees)	*P* value
Age (year)	70.8 ± 11.2	74.4 ± 8.5	0.051
Sex	M 18 F 82	M 28 F 72	0.130
BMI (kg/m^2^)	24.7 ± 4.4	26.3 ± 3.9	0.013
Height (cm)	153 ± 8.2	154 ± 8.9	0.289
HKA angle (degree)	7.81 ± 5.7	8.72 ± 5.0	0.060
	valgus	varus	
Coronal bowing angle (degree)	1.91 ± 2.8	3.38 ± 2.9	< 0.001
Sagittal bowing angle (degree)	10.5 ± 2.8	11.5 ± 2.6	0.002

Values are given as the mean and standard deviation

*Abbreviations*: *M* Male, *F* Female, *BMI* Body mass index, *HKA* Hip-knee-ankle

The DFVA in each DRR image and their difference relative to the NR and flexion in varus knees are presented in Table 2. The DFVA increased as the DRR image shifted from IR to ER. In each rotational position, the DFVA was significantly greater with 10° flexion. The DFVA in each DRR image and the difference relative to the NR and flexion in valgus knees are shown in Table 3. The DFVA increased as the DRR image shifted from IR to ER. The DFVA was significantly greater with 10° flexion in NR, 5° and 10° ER, but did not reach statistical significance in 5° and 10° IR.

**Table 2:** The distal femoral valgus angle in each DRR image and difference relative to the neutral flexion and rotation in varus knees

	IR 10	IR 5	NR	ER 5	ER 10
DFVA (degree) with neutral flexion	5.70 ± 1.57	6.07 ± 1.63	6.43 ± 1.63	6.68 ± 1.69	6.87 ± 1.73
Difference	-0.73 ± 0.40	-0.37 ± 0.25	0	0.24 ± 0.27	0.44 ± 0.38
DFVA (degree) with 10° flexion	5.78 ± 1.55	6.17 ± 1.62	6.52 ± 1.65	6.82 ± 1.71	7.03 ± 1.76
Difference	-0.65 ± 0.45	-0.26 ± 0.32	0.09 ± 0.24	0.39 ± 0.29	0.59 ± 0.43
*P* value	< 0.001	< 0.001	< 0.001	< 0.001	< 0.001

Values are given as the mean and standard deviation

*Abbreviations: DFVA* Distal femoral valgus angle, *IR* Internal rotation, *NR* Neutral rotation, *ER* External rotation

**Table 3:** The distal femoral valgus angle in each DRR image and difference relative to the neutral flexion and rotation in valgus knees

	IR 10	IR 5	NR	ER 5	ER 10
DFVA (deg) with neutral flexion	4.83 ± 1.60	5.11 ± 1.57	5.36 ± 1.60	5.57 ± 1.57	5.67 ± 1.55
Difference	-0.53 ± 0.39	-0.24 ± 0.33	0	0.21 ± 0.31	0.32 ± 0.33
DFVA (deg) with 10° flexion	4.86 ± 1.59	5.16 ± 1.56	5.46 ± 1.57	5.68 ± 1.59	5.81 ± 1.56
Difference	-0.50 ± 0.46	-0.20 ± 0.31	0.10 ± 0.26	0.32 ± 0.35	0.45 ± 0.39
*P* value	0.059	0.071	< 0.001	< 0.001	< 0.001

Values are given as the mean and standard deviation

*Abbreviations: DFVA* Distal femoral valgus angle, *IR* Internal rotation, *NR* Neutral rotation, *ER* External rotation

**Fig. 4:**
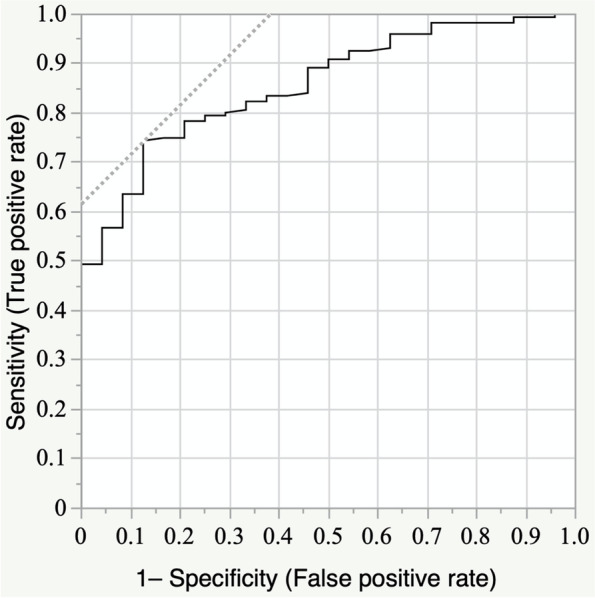
The receiver operating characteristic curve for large variation in the distal femoral valgus angle with sagittal femoral shaft bowing angle. The cut-off value of the sagittal bowing angle was 12.2° (sensitivity 74%, specificity 88%, area under the curve 0.86)

The variations of the DFVA with femur position are shown in Table 4. The DFVA variation was significantly greater in varus knees (Table 4). Eighteen knees (18%) in the varus group and six knees (6%) in the valgus group had a large variation (≥ 2°) of the DFVA (Table 5). After a univariate analysis of factors causing large variation, a factor of *p* < 0.20 was selected. Multivariate analysis showed that the sagittal femoral shaft bowing angle independently caused a large variation of the DFVA due to limb position (Tables 6 and 7). The results were similar for both varus and valgus knees. The receiver operating characteristic (ROC) curve determined that a sagittal bowing angle > 12° was associated with the large variation in the DFVA (sensitivity 74%, specificity 88%, area under the curve 0.86) (Fig. 4).

**Table 4:** Variation of the distal femoral valgus angle with femur limb position

	Varus	Valgus	*P* value
Variation of DFVA (degree)	1.46 ± 0.61	1.13 ± 0.51	< 0.001

Values are given as the mean and standard deviation

**Table 5:** Numeric distribution of Variation of the distal femoral valgus angle with femur limb position

		Varus (*n* = 100)	Valgus (*n* = 100)	*P* value
Variation of DFVA	≥ 2° (n)	18	6	0.009
	< 2° (n)	82	94	

*Abbreviations: DFVA* Distal femoral valgus angle

**Table 6:** Multivariate analysis of factors associated with the large variation (≥ 2°) of the DFVA due to limb position of the varus knees

Parameters	variation ≥ 2° (*n* = 18)	variation < 2° (*n* = 82)	Univariate *P* value	Multivariate *P* value
Age (year)	75.2 ± 9.1	74.2 ± 8.4	0.667	
Sex	M 2 F 16	M 26 F 56	0.058	0.456
BMI (kg/m^2^)	26.0 ± 4.0	26.4 ± 3.9	0.707	
Height (cm)	152.5 ± 8.4	154.9 ± 9.0	0.286	
HKA angle (degree)	9.5 ± 4.4	8.6 ± 5.1	0.442	
Coronal bowing angle (degree)	5.4 ± 3.2	2.9 ± 2.7	0.001	0.219
Sagittal bowing angle (degree)	13.8 ± 2.1	11.0 ± 2.8	< 0.001	0.001

*Abbreviations: BMI* Body mass index, *DFVA* Distal femoral valgus angle

**Table 7:** Multivariate analysis of factors associated with the large variation (> 2°) of the DFVA due to limb position of the valgus knees

Parameters	variation ≥ 2° (*n* = 6)	variation < 2° (*n* = 94)	Univariate *P* value	Multivariate *P* value
Age (year)	69.5 ± 9.8	70.9 ± 11.4	0.778	
Sex	M 3 F 3	M 15 F 79	0.064	0.922
BMI (kg/m^2^)	25.4 ± 3.5	24.7 ± 4.4	0.698	
Height (cm)	160.2 ± 8.0	152.8 ± 8.1	0.033	0.064
HKA angle (degree)	7.7 ± 7.6	7.8 ± 5.6	0.971	
Coronal bowing angle (degree)	2.1 ± 3.3	1.9 ± 2.8	0.829	
Sagittal bowing angle (degree)	14.4 ± 1.6	10.3 ± 2.7	< 0.001	< 0.001

*Abbreviations: BMI* Body mass index, *DFVA* Distal femoral valgus angle

## Discussion

Coronal whole-leg radiographs in NR are essential for evaluating the DFVA in preoperative TKA planning. Because radiographs are subjectively evaluated, the effects of whole-leg malrotation and knee flexion contracture on DFVA measurements are unclear. In addition, femoral shaft bowing might increase the measurement error. To the best of our knowledge, this is the first study analyzing this in detail in varus and valgus knees.

Judgment of “neutral rotation” in the whole-leg radiograph is subjectively made by surgeons based on the “patellar neutral” position. However, Kawakami et al. reported that whole-leg radiographs taken in the “patellar neutral” position ranged from 8° ER to 14° IR [6]. Kawahara et al. also showed that DRR images from NR to 10° IR relative to the SEA could be judged as neutral whole-leg radiographs [13]. In addition to variations in subjective judgments of "neutral rotation", hip and ankle deformities, obesity, and swelling of the knee joint have also been reported to cause malrotation [14]. To simulate the variation in measurements due to the lower limb rotation, the coronal plane was set parallel to the SEA in order to define a criterion with eliminated subjectivity [6, 13]. Moon et al. investigated the correlation between lower limb rotation and radiographic parameters by assessing the medial or lateral deviation of the patella relative to the femoral condyle [9]. Quantifying malrotation using radiographic parameters may further improve the accuracy of preoperative planning.

The DFVA increased as the DRR image shifted from IR to ER, and all angles increased further from extension to 10° flexion in varus and valgus knees. A study using synthetic femur and tibia bone models also reported the same tendency [15]. The DFVA increases as the lower extremity is externally rotated because the distal femoral anatomical axis is inclined laterally [16]. Therefore, surgeons need to understand how rotation affects DFVA measurement.

Further, no study has evaluated the effects of knee flexion contracture on these measurements. The DFVA was proved to increase with knee flexion contracture. When the femur is in flexion, the femoral mechanical axis is projected shorter, whereas the mediolateral width of the femur is projected as the same in the coronal plane. Consequently, the angle between the mechanical axis and the distal anatomical axis is relatively greater, which is considered to increase DFVA. Previous reports have reported greater variability in DFVA measurements when malrotation is combined with knee flexion [17]. Therefore, surgeons need to be cautious in judging the rotation of the whole-leg radiograph, especially in cases with knee flexion contracture.

On average, the variation of DFVA in each femur limb position was 1.3° (1.5° for varus and 1.1° for valgus knees). Further, 18% of varus and 6% of valgus knees showed a variation of 2° or more. A postoperative HKA angle within 3° of neutral alignment was reported to reduce the revision rate and improve patient function [11]. Assuming that the distal femoral resection is adjusted in 1° increment using an intramedullary rod, cut-off values were determined based on previous reports [10, 11]. Sikorski et al. reported that it is desirable and achievable to place components within 2° of neutral in the femur and tibia, respectively [10]. A measurement variation of 2° or more caused by limb malposition during preoperative planning could be large enough to cause postoperative malalignment of over 3°.

Understanding the femoral morphology was also important in preoperative planning. The effect of coronal and sagittal femoral shaft bowing on DFVA measurement in the presence of malrotation has not been well reported. In this study, the multivariate regression analysis showed that sagittal femoral shaft bowing was independently associated with large variations of the DFVA with each limb position. In cases with large sagittal bowing, the distal anatomical axis would be laterally inclined to a greater extent as the femur is externally rotated, which would result in larger variations in measurements. The variation of the DFVA was greater in varus knees, which can be explained by the fact that the sagittal bowing angle was greater. Moreover, our ROC analysis showed that more than 12° of sagittal bowing caused the large variation of the DFVA between limb positions. It had a good area under the curve, sensitivity, and specificity. In all, 34% of all cases (40%, varus and 27%, valgus) had 12° or more sagittal femoral shaft bowing. In these cases, close attention should be paid to evaluating the malposition of the lower limb when taking the whole-leg radiograph. Preoperative planning with CT data is recommended.

Previous studies have reported that in patients with large sagittal bowing, the distal femoral anatomical axis is more flexed relative to the femoral mechanical axis, so TKA using an intramedullary rod is more likely to result in femoral component flexion [5, 18]. Angle calibration can be achieved by altering the intramedullary rod entry point (more anterior than the routine) or using the navigated TKA system [5, 18] and extramedullary reference [19]. The flexed placement of the femoral component greater than 3.5° in the sagittal plane is an independent risk factor for clinically detectable flexion contracture [20]. Flexion contractures are reported to cause shortening of the effective leg length [21], leading to limping, decreased walking speed, and contralateral flexion contractures [22, 23]. In cases of large sagittal femoral shaft bowing, clinical symptoms such as flexion contractures could be prevented by avoiding flexed placement.

The strength of this study is that much CT data could be acquired and analyzed in detail, especially in valgus knees. Previous anatomical or image analysis of whole-leg CT data of valgus knees included 5 to 63 knees [24–26]. To the best of our knowledge, our study has the most whole-leg CT data of valgus knees. This study has some limitations. First, this is an image analysis study of the effects of specific factors on DFVA measurement in TKA preoperative planning. However, postoperative component placement angles and clinical outcomes, including long-term outcomes, were not evaluated. Second, this study population was limited to Japanese subjects. Japanese and Caucasians have several anatomical differences [27]. As femoral shaft bowing is more common in Asians, the results may not generalize to different races. Third, the present study included 22 patients diagnosed with rheumatoid arthritis (24 valgus knees and 2 varus knees). Morphology may be changed in patients with rheumatoid arthritis. A multivariate regression analysis was also performed on 24 valgus knees with rheumatoid arthritis, and similarly, sagittal femoral shaft bowing was independently associated with a large variation of DFVA (*p* = 0.027). The main results of this study are consistent for rheumatoid arthritis cases, and the same attention should be paid in clinical practice. However, the valgus knee is rare. By including consecutive cases of knee arthroplasty, the study was more reflective of daily clinical practice. Fourth, we assumed 20° of knee flexion contracture with 10° femoral flexion. We could not perform the simulation with positioning that strictly assumes knee flexion contracture, but we performed simulations assuming various malpositions by combining femoral flexion and internal and external rotation.

## Conclusions

During TKA preoperative planning with whole-leg radiographs, the DFVA was greater with the limb in external rotation and with flexion contracture. Sagittal femoral shaft bowing was independently associated with large variations of the DFVA. If the femoral sagittal bowing angle is more than 12°, close attention should be paid to the lower limb position of the radiographs. Further, preoperative planning with whole-leg CT data should be considered.

## Data Availability

The datasets generated during and analyzed during the current study are not publicly available due to their containing information that could compromise the privacy of research participants but are available from the corresponding author on reasonable request.
